# RSYD-BASIC: a bioinformatic pipeline for routine sequence analysis and data processing of bacterial isolates for clinical microbiology

**DOI:** 10.1099/acmi.0.000646.v6

**Published:** 2025-03-21

**Authors:** Kat Steinke, Karina Gravgaard Thomsen, Silje Vermedal Hoegh, Sanne Løkkegaard Larsen, Karina Kubel Vilhelmsen, Thøger Gorm Jensen, Marianne Nielsine Skov, Thomas Vognbjerg Sydenham

**Affiliations:** 1 Department of Clinical Microbiology, Odense University Hospital, Odense, Denmark; 2 Research Unit of Clinical Microbiology, Faculty of Health, University of Southern Denmark, Odense, Denmark

**Keywords:** bioinformatics, clinical microbiology, whole-genome sequencing

## Abstract

**Background.** Whole-genome sequencing of bacterial isolates is increasingly becoming routine in clinical microbiology; however, subsequent analysis often needs to be started by a bioinformatician even for comprehensive pipelines. To increase the robustness of our workflow and free up bioinformatician work hours for development and advanced analysis, we aimed to produce a robust, customizable bioinformatic pipeline for bacterial genome assembly and routine analysis results that could be initiated by non-bioinformaticians.

**Results.** We introduce the RSYD-BASIC pipeline for bacterial isolate sequence analysis and provide a demonstration of its functionality with two datasets composed of publicly available sequences, in which comparable results are obtained in most cases. In some instances, the pipeline provided additional information, corresponding to *in vitro* results where these could be obtained. In routine use at our department, the pipeline has already yielded clinically relevant results, allowing us to type a variety of bacterial pathogens isolated in our clinical laboratory. We also demonstrate how RSYD-BASIC results aided in disproving a potential outbreak.

**Conclusion.** With the RSYD-BASIC pipeline, we present a configurable reads-to-results analysis pipeline operated by non-expert users that greatly eases the investigation of potential outbreaks by expert end users. Results obtained with publicly available sequences show comparable performance to the original methods, while underlining the importance of standardized methods.

## Data Summary

The code of the RSYD-BASIC pipeline is available at https://gitlab.com/KatSteinke/rsyd-basic.

GenBank accession numbers and/or PubMLST identifiers of sequences used for the test datasets and the example of combining RSYD-BASIC results with manual investigation are listed in the ‘Methods’ section.

The small demonstration dataset (reads and metadata files) and analysis results for this dataset are available on Zenodo at https://zenodo.org/records/13881353. The dataset derived from a public antimicrobial resistance benchmarking dataset and analysis results for this dataset are available on Zenodo at https://zenodo.org/records/13889977.

The patient sequences examined in the outbreak investigation are uploaded to the NCBI as assemblies GCF_046765375.1 (patient 1) and GCF_046765395.1.

## Introduction

Many view next-generation sequencing of bacterial isolates as an important future tool in clinical diagnostics [1,2]. For example, whole-genome sequencing (WGS) of bacterial isolates can be used routinely for typing of pathogens including identification of transmission of multi-drug-resistant bacteria [3,4], predicting pathogen serotypes [5], improving species identification with possible clinical benefits [6] and identifying antimicrobial resistance (AMR) genes and to some extent predicting AMR [7]. However, bioinformatic analyses are required to process and interpret the vast amounts of data generated.

A number of open-source pipelines that can perform ‘reads-to-results’ analyses exist, as do commercial options such as Illumina’s BaseSpace suite or the 1928 Platform (1928 Diagnostics). These examples can require the upload of sequence data, which is not always practically preferable in regard to patient data privacy. Commercial stand-alone solutions such as SeqSphere+ [8] exist, but often, there will be local-specific needs for tool implementations and the development of in-house solutions to aid analysis. Bioinformatic analysis of genome sequencing data is a fast-evolving field. Therefore, laboratories frequently choose to implement their own bioinformatic solutions. Open-source tools that can run locally, such as Bactopia [9], often require some familiarity with running programmes from the command line. This can pose a challenge for laboratory technicians. Therefore, starting the analysis pipeline often depends on a bioinformatician (or, depending on personnel resources, *the* bioinformatician) or other personnel with knowledge of computational biology, which makes the process considerably less robust. The combined output from the individual tools in such pipelines may be difficult to interpret [1], and from an operational perspective, building solutions that create outputs tailored for import into the local laboratory information systems (LISs) can be preferable.

We aimed to develop and implement a customizable user-friendly open-source pipeline to allow analysis of bacterial WGS data in a clinical microbiology laboratory.

## Methods

### Workflow description

**Fig. 1.**
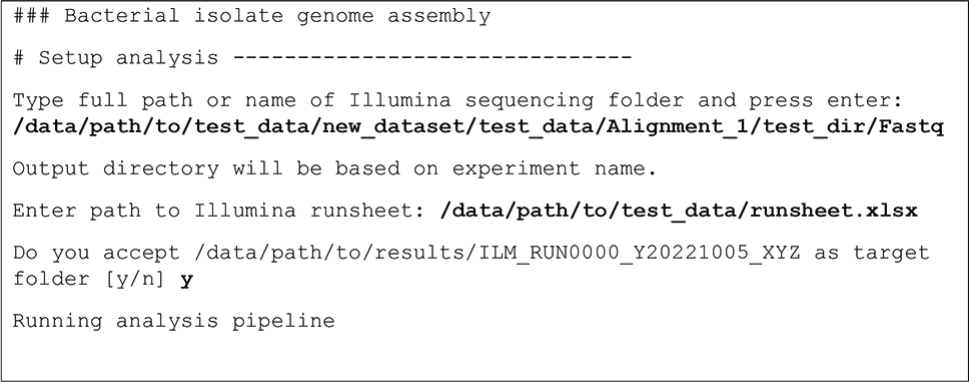
Example of the pipeline’s ‘questionnaire’ mode with a test dataset, showing the pipeline’s prompts and the user’s input. User input is bolded for clarity in this only; this does not represent a feature of the pipeline.

The RSYD-BASIC (Routine Sequence analYsis and Data processing of BActerial iSolates for clInical miCrobiology) pipeline, once set up by a bioinformatician, can be started in two different ways: for routine uses, a ‘questionnaire’-style interface in the terminal guides through which files need to be supplied (see Fig. 1 for an example), with most settings predefined through a default configuration file. For more experienced users, the pipeline can be launched through the command line as well, allowing for more fine-grained configuration.

As input, the pipeline takes a directory containing Illumina or Nanopore reads from multiple bacterial isolates from one sequencing run in fastq format, as well as a ‘run sheet’ with metadata for the run, including indications for sequencing (for detailed requirements, see the pipeline’s repository). Column and sheet names of the metadata sheet can be specified in a separate config file (‘input config file’); sample input config files for Danish and English input can be found in the repository. An English version of the metadata sheet can be found in the supplementary data on Zenodo (https://zenodo.org/records/13881353).

Note that this pipeline is not suited for the analysis of metagenomic data or non-bacterial sequences.

A report from the LIS can be supplied as well, enabling cross-checking of sample numbers and supplied species identification; the location of this report is specified through the configuration file. Column names for this report can be specified in the input config file as well. An example with explanations is given as Table S1 (Note that this supplementary file cannot be used as direct input to the pipeline, as the pipeline expects the LIS report to be a semicolon-separated file; an LIS report that can be used for analysis of the test set is available in the dataset deposited on Zenodo.) If LIS data is stored in an SQL database, the pipeline can be configured to retrieve the information and collate the report itself.

**Fig. 2.**
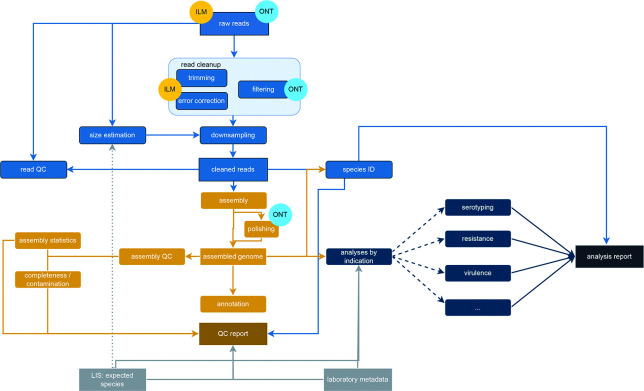
The data flow in the RSYD-BASIC pipeline. Light blue boxes represent operations performed and data obtained from raw reads; golden boxes represent operations performed on assemblies; dark blue boxes represent specific analyses based on indication; grey boxes represent external data sources. The darkest golden and blue boxes represent the analysis results for the respective inputs. Illumina- and Nanopore-only steps are marked with bubbles labelled ‘ILM’ and ‘ONT’ respectively.

The analysis workflow, shown in Fig. 2, takes inspiration from other reads-to-results pipelines such as Bactopia and is implemented using Snakemake [10].

For Illumina, read sequences for each sample are initially cleaned by removing the sequencing adapter and any reads belonging to the PhiX sequencing control with bbduk version 38.96 [11]. Errors are then corrected with lighter [12]. From the raw reads, genome size is estimated using Mash version 2.3 [13]; this estimate is then used to downsample the cleaned, corrected reads to a maximum of 100× coverage [14], if required, using reformat.sh from version 38.96 of the BBTools suite [11]. Finally, quality control reports on the reads before and after cleaning are obtained using FastQC [15].

For Nanopore reads, the pipeline will attempt to leverage species identity information from the LIS to obtain estimates for genome size if present, as kmer-based estimates may yield overly large genome sizes for Nanopore data due to the higher error rates. As an additional mitigation step, if Mash-based genome size estimation is necessary, it is performed after a filtering pass with filtlong version 0.2.1 [16] that removes all reads shorter than 1000 bp and retains at most the 95% best reads. After an estimated genome size is obtained, downsampling is performed using filtlong again to retain the best reads up to a coverage of 100×.

The cleaned reads are also analysed with version 2.1.2 of Kraken2 [17] with a user-specified database [for our use case at the Department of Clinical Microbiology, Odense University Hospital (DCM OUH), the Standard-8 database, version 12/9/2022]. This may support the investigation of possible contamination later in the process, as the Kraken output shows the proportion of reads belonging to each organism. Isolate sequences are also compared to databases of RefSeq [18] sequences using Mash version 2.3 [13] and sourmash version 4.4.0 [19].

Subsequent analyses (shown as golden boxes in Fig. 2) are performed on assemblies. For Illumina, these assemblies are obtained using shovill version 1.1.0se [20], with skesa version 2.4.0 as the assembler. For Nanopore, flye version 2.9.3 [21] is used. Nanopore assemblies on the basis of high-accuracy reads are polished with medaka (version 1.11.3) [22], while no polishing is performed on super-accuracy reads due to findings, suggesting that polishing may worsen the quality for these reads [23]. We do not recommend using reads obtained with fast basecalling for the pipeline.

General quality control metrics such as N50, NG50, genome size and amount of contigs are obtained using QUAST version 5.2.0 [24]. Completeness and contamination are estimated using CheckM version 1.2.3 [25]. Finally, species identification is performed using the classify_wf workflow of GTDB-Tk version 2.3.2 [26] and a user-specified database (at DCM OUH, this is release 214 of the GTDB database). Briefly, this attempts to classify samples based on Mash-estimated average nt identity to GTDB reference genomes, and where this is not possible, placement of the sample in the GTDB-Tk reference tree based on a concatenated multiple sequence alignment of a set of reference genes. Quality control (QC) results as well as the GTDB-Tk species call and the species for which the highest proportion of reads match in Kraken are reported in a QC-focused result file. The format of this file has been developed in close cooperation with the molecular biologists at DCM OUH. If an LIS report has been supplied, any preliminary species ID registered in the LIS as well as the expected genome size for this species (if available) will be shown in the QC results for comparison to aid with the detection of laboratory errors.

In addition, possible clinically relevant properties of the isolates are investigated. By default, this currently encompasses

- AMR genes (using abritAMR 1.0.14 [27])
- Plasmids (using PlasmidFinder 2.1.6 [28])
- MLST typing (using mlst 2.16.1 [29,30])

If requested in the run sheet, selected virulence or toxin genes can also be reported. These are identified from abritAMR’s output as well.

Genomes are annotated using Prokka version 1.14.6 [31].

Species-specific analyses are currently performed only when an LIS report is given. Currently, the only species-specific analyses are serovar identification for *Salmonella* (using SeqSero2 version 1.3.1 [32] on cleaned reads for Illumina data and assemblies for Nanopore) and serotyping for *Escherichia coli* (using SerotypeFinder version 2.0.1 [33]).

These results are then compiled into an analysis result file. For the analysis report file, results from the tools used can be filtered tailored to the use case of the user. For example, at our department, we have decided to only include abritAMR results on beta-lactams and colistin resistance determinants; full abritAMR results can of course still be accessed if required. Result files can be output in Danish or English, with language specified in the pipeline’s config file.

Finally, QC results are evaluated – this is partially automated for several ‘hard’ cutoffs, but some results may require manual examination. Cutoffs are explained in Table 1.

After QC evaluation, analysis results may be used further in routine procedures.

**Table 1.** QC parameters and actions taken

QC value	Automated evaluation?	Required value and response if value differs	Source
Average coverage	Yes	Illumina: ≥30×Nanopore: ≥60×If coverage is lower, sequence is rejected	Illumina: [58]Nanopore: own comparison with Illumina data
N50	Yes	≥10 000If N50 is lower, sequence is rejected (highly fragmented data)	
Genome size	Yes	Genome size ≥80% and ≤120% of expected organism’s genome sizeIf genome is smaller or larger, sequence is rejected (could be incomplete data if smaller, contamination if larger, wrong sample in both cases)	
CheckM contamination	No	Contamination ≥1% triggers investigation (e.g. GC content of reads for multiple ‘peaks’, Kraken for large proportion of reads matching another species); may be legitimate for some species	
Amount of contigs	No	For Nanopore assemblies, amount of contigs >10 triggers investigation (assembly visualization: is the chromosome closed?)	

#### Acquisition of test datasets

##### Demonstration dataset

Existing publicly available reads for species of interest were downloaded from the NCBI’s Sequence Read Archive (SRA) [34] in SRA Normalized Format (preserving quality scores), using version 2.10.0 of the SRA toolkit [35]. Read accessions and sample numbers assigned in the test dataset are shown in Table 2.

**Table 2.** Reads used in the demonstration dataset

Sample no.	SRR accession no.	Species	Source
1199234567-1	ERR9793822	*Salmonella* Newport	[59]
1199234567-2	SRR7235142	*Escherichia coli*	[60]
1199234567-3	SRR14194623	*Klebsiella pneumoniae*	[39]
1199234567-4	SRR10955980	*Streptococcus pneumoniae*	[61]
1199234567-5	SRR10955980	*Streptococcus pneumoniae*	[61]

To provide a deliberately ‘failed’ sample (sample 1199234567-5), 1000 forward reads and 1000 reverse reads were sampled from read set SRR10955980 using seqtk sample [36].

##### Larger dataset (subset of AMR benchmarking dataset)

The larger evaluation dataset was based on a publicly available benchmarking dataset [37]. The dataset’s metadata was obtained, and SRR accession numbers for the respective reads were identified. As different Illumina sequencers require slightly different settings in the pipeline, the dataset was filtered, so only paired-end MiSeq reads remained. The remaining 29 sequences, shown in Table 3, were downloaded using version 2.10.0 of the SRA toolkit in SRA Normalized Format. Two samples (SRR1284629 and SRR1501122) were subsequently removed from analysis as the NCBI Assembly record marked a mismatch between species given on upload and species found by the NCBI’s species verification workflow [38] for the assemblies obtained from the respective reads.

**Table 3.** Reads from the AMR benchmarking dataset

Sample no.	SRR accession no.	Species	Additional metadata source
1199123456-1	SRR3465535	*Aeromonas veronii*	[62]
1199123456-2	SRR1284629	*Citrobacter freundii*	[63]
1199123456-3	SRR1576808	*Enterobacter hormaechei*	[64]
1199123456-4	SRR1576778	*Enterobacter hormaechei*	[64]
1199123456-5	SRR2120261	*Enterococcus faecium*	[52]
1199123456-6	SRR3999067	*Escherichia coli*	
1199123456-7	SRR2533563	*Escherichia coli*	[65]
1199123456-8	SRR3999064	*Escherichia coli*	
1199123456-9	DRR065947	*Escherichia coli*	[66]
1199654321-1	SRR3931165	*Escherichia coli*	[67]
1199654321-2	SRR2014925	*Escherichia coli*	[68]
1199654321-3	SRR1501122	*Klebsiella oxytoca*	[69]
1199654321-4	SRR3222213	*Klebsiella pneumoniae*	
1199654321-5	SRR1510963	*Klebsiella pneumoniae*	[64]
1199654321-6	SRR2724081	*Klebsiella pneumoniae*	
1199654321-7	SRR3219268	*Klebsiella pneumoniae*	
1199654321-8	SRR1427234	*Klebsiella pneumoniae*	[64]
1199654321-9	SRR2244257	*Klebsiella pneumoniae*	[70]
1199345678-1	SRR3709728	*Salmonella* Heidelberg	[71]
1199345678-2	SRR2598330	*Salmonella* Heidelberg	[72]
1199345678-3	SRR3707412	*Salmonella* Heidelberg	[73]
1199345678-4	SRR3707415	*Salmonella* Heidelberg	[74]
1199345678-5	SRR2896022	*Salmonella* Heidelberg	[72]
1199345678-6	SRR3707447	*Salmonella* Heidelberg	[75]
1199345678-7	SRR2898679	*Salmonella* Heidelberg	[72]
1199345678-8	SRR2407791	*Salmonella* Infantis	[76]
1199345678-9	SRR1395326	*Salmonella* Minnesota	[77]
1199876543-1	SRR2939562	*Salmonella* Infantis	[78]
1199876543-2	SRR3173804	*Salmonella* Weltevreden	[79]

### Evaluation of results

#### Demonstration dataset

Results were compared against those found in the articles originally describing the sequences. Where a discrepancy was found, this was investigated using the original assembly used in the article. This was only the case for *Klebsiella pneumoniae*. Both the RSYD-BASIC assembly and the original assembly (GCA_018138665.1) were analysed using both the tool used in RSYD-BASIC (mlst [29,30]) and the tool used by Fursova *et al*. [39] (MLST [40]). For the latter, the online version of the tool available at https://cge.food.dtu.dk/services/MLST/, which used version 2.0.9 at the time of analysis, was used. This was also used to analyse the raw reads. The database in use was the most current database at the time of analysis (version 2023-06-19).

#### Larger dataset (subset of AMR benchmarking dataset)

AMR results were compared against the results in the public benchmarking dataset [37], taken from https://github.com/AMR-Hackathon-2021/benchmarking_datasets/blob/main/data/results.zip. Only those AMR gene classes that are reported in the final report file by RSYD-BASIC were considered. The code and files used for gene class filtration are available at https://gitlab.com/-/snippets/3756907. Where available, additional information (e.g. on serovar, serotype or sequence type (ST)) was taken from the articles originally describing the samples, or the NCBI’s sample information (given as ‘additional metadata source’ in Table 3).

To examine the effects of the AMR gene prediction tool on results, the assemblies generated with RSYD-BASIC were also analysed with CARD-RGI version 5.2.0 [41] at default settings using version 3.1.4 of the CARD database [41], as in the original benchmarking analyses [37]. Raw results for this are available as Data S1 on https://zenodo.org/records/13923953. Commands run are shown in the ‘running CARD-RGI on RSYD-BASIC assemblies’ section of File S 1. Where it was necessary to compare AMR gene sequences found by CARD-RGI in the original and RSYD-BASIC sequences either to each other or to current sequences in the CARD, this was done with Needleman–Wunsch alignments of the predicted DNA sequence with EMBOSS Needle through the EMBL-EBI job dispatcher [42]. The resulting alignments are available as Data S2 on https://zenodo.org/records/13923953.

### Sequencing statistics

The amount of samples sequenced and their indications were extracted in R using the tidyverse package [43].

### Manual outbreak investigation

All isolates with the same MLST type as identified by mlst in the RSYD-BASIC pipeline were analysed further with ChewBBACA version 2.8.5 [44] using the *Staphylococcus aureus* cgMLST scheme of Leopold *et al*. [45], processed with ChewBBACA’s PrepExternalSchema function. For comparison, unrelated sequences from PubMLST [46] with the same sequence type were added (see Table 4). Alleles were called with chewBBACA AlleleCall with default parameters and the prepared scheme, and the results were cleaned with chewBBACA ExtractCgMLST (setting the threshold for evaluating whether a gene is part of the core genome to 0 so that the script only removed ‘INF-’ prefixes and set remaining non-integer allele values to 0).

**Table 4.** *Staphylococcus aureus* sequences used as background for cgMLST

Database ID	ST	Database	URL
42320	45	PubMLST	https://pubmlst.org/bigsdb?page=info&db=pubmlst_saureus_isolates&set_id=1&id=42320
41852	45	PubMLST	https://pubmlst.org/bigsdb?page=info&db=pubmlst_saureus_isolates&set_id=1&id=41852
41843	45	PubMLST	https://pubmlst.org/bigsdb?page=info&db=pubmlst_saureus_isolates&set_id=1&id=41843
42318	1	PubMLST	https://pubmlst.org/bigsdb?page=info&db=pubmlst_saureus_isolates&set_id=1&id=42318
39207	1	PubMLST	https://pubmlst.org/bigsdb?page=info&db=pubmlst_saureus_isolates&set_id=1&id=39207
39450	1	PubMLST	https://pubmlst.org/bigsdb?page=info&db=pubmlst_saureus_isolates&set_id=1&id=39450
39288	30	PubMLST	https://pubmlst.org/bigsdb?page=info&db=pubmlst_saureus_isolates&set_id=1&id=39288
41833	30	PubMLST	https://pubmlst.org/bigsdb?page=info&db=pubmlst_saureus_isolates&set_id=1&id=41833
41841	30	PubMLST	https://pubmlst.org/bigsdb?page=info&db=pubmlst_saureus_isolates&set_id=1&id=41841

A minimum spanning tree was then constructed using the goeBURST Full MST functionality in PhyloViz 2.0 [47].

SNP analyses were performed on ST1 samples using snippy’s snippy-multi functionality [48]. Sequences were supplied as assemblies, and the *Staphylococcus aureus* sequence with pubMLST database ID 39207 was used as the reference sequence as it appeared to possess roughly equal distance to the other sequences. cgMLST allele difference and SNP difference cutoffs given by Schürch *et al*. [49] were used as a basis for the evaluation of relatedness.

The precise commands are available in File S2. cgMLST profiles for the ST1 patient and background samples are available as Table S4. Sequences for samples from patients 1 and 2 are uploaded to the NCBI in assemblies GCF_046765375.1 and GCF_046765395.1, respectively.

### Results and discussion

#### Example output

A small basic test set compiled from publicly available Illumina MiSeq reads is available to users to demonstrate the pipeline’s functionality. The test set consists of *Salmonella* Newport to test serovar detection, *Escherichia coli* for serotyping, *K. pneumoniae* for resistance and plasmid detection, a *Streptococcus pneumoniae* sample and a deliberately ‘bad’ sample generated by subsampling the *Streptococcus pneumoniae* sample.

Key results for the samples are shown and compared with known results in Table 5.

**Table 5.** Comparison of RSYD-BASIC results to original results for a publicly available demonstration dataset

Sample no.	RSYD-BASIC species call	RSYD-BASIC serovar/serotype	RSYD-BASIC MLST	RSYD-BASIC toxin genes	Original species call	Original serovar/serotype	Original MLST	Original toxin genes
1199234567-1	*Salmonella enterica*	Newport	46		*Salmonella enterica*	Newport	46	
1199234567-2	*Escherichia coli*	O22:H8	446	STX1A, STX2C	*Escherichia coli*	O22:H8		*stx2*
1199234567-3	*K. pneumoniae*		395		*K. pneumoniae*		2674*	
1199234567-4	*Streptococcus pneumoniae*		416		*Streptococcus pneumoniae*		416	
1199234567-5	na	na	na	na	*Streptococcus pneumoniae*		416	

* ST reported in article; ST obtained from deposited sequence data: 395.

The results obtained with the RSYD-BASIC pipeline generally agree with those found in the articles initially describing the sequences analysed, with two differences. Firstly, no results are obtained for 1199234567-5; this is to be expected, as it deliberately simulates a bad sample. Secondly, the MLST type of the *K. pneumoniae* sample does not match that given in the original article. However, the two types differ only by a single allele [46], and reanalysis of the original assembly deposited in the NCBI’s database with both the mlst tool [29] in the RSYD-BASIC pipeline and the currently available version of the MLST tool [40] used by Fursova *et al*. [39] in the original article (version 2.0.9, using database version 2023-06-19) yielded MLST type 395 as well. However, the Center for Genomic Epidemiology’s MLST tool also allows the use of reads. Therefore, MLST analysis was repeated using the original reads. The MLST type reported for analysis of the reads was also MLST type 395.

The results seen with the test dataset using RSYD-BASIC are generally comparable to those found in the articles describing the original sequences, which were obtained with a range of different methods. However, the *K. pneumoniae* sample has a different MLST type than the one originally given in the literature. The two types differ by a single allele, with the difference between the two alleles being a single nt [46]. Any difference in read filtering, error correction or assembly may therefore have caused the discrepancy – this may also explain the difference between the publicly available sequence (assembled using unicycler) and that used in the original article by Fursova *et al*. (assembled using SPAdes). This underlines the importance of standardized analysis methods to ensure consistent results.

The full result files can be found in Tables S2 (QC report) and S3 (analysis report) of . The original outputs are available at https://zenodo.org/records/13881353.

#### Larger dataset (subset of AMR benchmarking dataset)

A subset of a larger publicly available dataset used for benchmarking of AMR gene detection was used for a larger-scaled demonstration. The original outputs are available at https://zenodo.org/records/13889977. As shown in Table 6, the majority of results that could be compared matched: species, *Salmonella* serovars and ST types, where given, were identical between RSYD-BASIC and the original data associated with the dataset.

**Table 6.** Overview of matches/mismatches between RSYD-BASIC results and available information for the dataset drawn from a publicly available AMR benchmarking dataset Alongside the total amount of mismatches, the amount of these in which results partially match and the amount where the difference was deemed to be clinically relevant is shown

Result category	Match	Mismatch (total)	Mismatch (clinically relevant)*	Mismatch (partial match)
Species	27	0	0	0
Serovar/serotype	12	3	3	3
ST type	7	0	0	0
Toxin genes	1	1	0	1
Filtered AMR genes	0	26	1†	13‡

* ‘Clinically relevant’: mismatches that potentially could impact treatment or outbreak monitoring, according to our assessment.

† Additional mutation potentially conferring beta-lactam resistance found by abritAMR in RSYD-BASIC.

‡ ‘Partial match’ for AMR genes: RSYD-BASIC results are a subset of original results or vice versa.

For *Escherichia coli*, where serotype information was present, H type matched in all cases, while O type could not be identified in two cases and did not match in others. In a third, RSYD-BASIC identified the serotype as O18ab:H14, while the NCBI sample metadata gave the serotype as O18ac:H14. While it was unclear how the serotype was identified for the NCBI sample metadata, it is relevant to note that SerotypeFinder is unlikely to be able to distinguish between the two as the variants are highly identical [33].

All specified toxin genes were found. Stx types given by RSYD-BASIC matched those given in the original articles. It must be noted that RSYD-BASIC, on account of using abritAMR, provided more detailed subtyping data, which could not be found for the original sequences, but which in one case would have impacted treatment. In one case, RSYD-BASIC additionally identified binary toxin.

Filtered AMR gene data partially matched the results determined with CARD-RGI for the benchmarking dataset. To investigate whether the discrepancies were due to differences between the tools used or differences in the assemblies, assemblies produced by RSYD-BASIC were examined with the same software and database version of CARD-RGI as the one used in the benchmarking dataset, using default settings. Results are available as File S1 at https://zenodo.org/records/13923953.

In the majority of cases, CARD-RGI results matched completely. We can therefore conclude that tool choice was responsible for the majority of the discrepancies. While most genes missing in the abritAMR output were found with both sets of assemblies, suggesting the difference in tools as the cause, CARD-RGI also found some short fragments of genes in the assemblies produced by RSYD-BASIC. This is likely due to these assemblies being fragmented, while the original assemblies were closed. abritAMR, meanwhile, would not report such fragments, as they are all shorter than its default coverage cutoff of 50%. Similarly, one gene only found with CARD-RGI (in both sets of assemblies) only had 31% coverage and likewise would not be reported.

Differences in defaults also may explain another gene found with CARD-RGI. This gene, *Haemophilus influenzae* PBP3, was called on the basis of hits with ~50% identity, considerably lower than abritAMR’s default requirement of 90% identity used in the pipeline. Along with two other genes found in almost all CARD-RGI results – ‘*Escherichia coli* ampH beta-lactamase’ and ‘*Escherichia coli* ampC1 beta-lactamase’, which were removed in the later version 3.2.3 of the database [50] – this accounted for about half of the mismatches between abritAMR and CARD-RGI.

Similarly, one discrepancy may have stemmed from the programmes’ different default requirements for coverage, with CARD-RGI reporting a gene with 31% coverage while abritAMR, which requires 50% by default, did not.

Finally, even when both programmes reported the same match to identical proteins, interpretation could differ – a 97.88% match to a blaEC-8 sequence was reported as blaEC-8 by CARD-RGI and unspecified blaEC by abritAMR.

One notable instance where CARD-RGI results differed from each other was for sequences in which the original results showed partial hits for the first half of blaOXA-896 and the second half of blaOXA-9, separated by a few hundred bp on the same contig. This was the case for samples 1199123456-8, 1199654321-5 and 1199654321-8. Here, running CARD-RGI on the assemblies produced by RSYD-BASIC instead resulted in two halves of blaOXA-9 being found. In the abritAMR results, meanwhile, these were interpreted as a single hit, matching blaOXA-9 with an internal stop codon. A similar situation led to a sequence being reported as a blaEC gene with an internal stop codon by abritAMR and two partial genes (blaEC-8 and blaEC-16) by CARD-RGI for sample 1199123456-9.

Additionally, in several cases, CARD-RGI predicted the presence of blaSHV-66 in assemblies produced by RSYD-BASIC, while the original CARD-RGI results identified an identical sequence as blaSHV-134. As settings for the original CARD-RGI results obtained for the reference dataset were not reported, it is not clear whether this arose due to a difference in settings. abritAMR, meanwhile, identified the gene as blaSHV-12 in all cases; it should be noted that the version of the database used to obtain CARD-RGI results only contained an incomplete version of the blaSHV-12 sequence [51]. The sequence found in the version of the CARD at the time of writing (version 3.3.0), meanwhile, is an exact match to the sequences found. Alignments are available as Data S2; files named *_rsyd_basic_vs_original.out compare sequences found in the RSYD-BASIC assemblies to the originals, while files named *_rsyd_basic_vs_card_shv-12.out compare sequences from the RSYD-BASIC assemblies to the SHV-12 DNA sequence available in version 3.3.0 of the CARD.

Conversely, abritAMR was able to report a number of point mutations, which were not reported by CARD-RGI. While these require expertise to interpret, they did in one case (highlighted in Table 6) correspond to the AMR profile reported in the respective study: sample 1199123456-5, an *Enterococcus faecium* sample with a point mutation conferring potential beta-lactamase resistance reported in abritAMR, was indeed shown to possess beta-lactam resistance in the original study by Fiedler *et al*. [52].

The difference in AMR genes reported underlines the impact of users’ choice of software, settings and databases on results, as well as the necessity of documenting each of these for reproducibility.

These comparisons also underscore the difficulty of validating bioinformatic pipelines: we do not always know the ‘correct’ results and can only compare against the data that are available. Generally, however, we conclude that RSYD-BASIC produces useful results: the majority of comparable results match those found in the original studies, and for AMR genes, where the largest discrepancies were seen, the only potential clinically relevant change was one in which RSYD-BASIC results reflected experimental results.

However, it must be noted that *Escherichia coli* O antigens could not be identified in several cases with the present dataset. In some cases, no O antigen sequence was found, while in one, the pipeline could not differentiate between O18ab and O18ac. This may pose an issue, especially as O antigen terminology is commonly used in outbreak notifications. Missing or incorrect O antigen information may therefore delay outbreak detection. However, routine surveillance measures may mitigate this to some extent. At our department, for instance, new cgMLST trees are generated (see ‘Manual outbreak investigation’) for all species for which new outbreak, relapse or potential healthcare-associated infection sequences were obtained after each sequencing run; if multiple outbreak samples were sequenced or an external outbreak sequence was available for comparison, the sequences would nevertheless cluster and raise suspicion.

While the immediate results certainly require expertise to interpret and awareness of limitations is necessary, RSYD-BASIC nevertheless may be a useful tool for clinical microbiology.

#### Real-world application

At our department, we have defined categories of isolates that are referred to WGS. Examples include isolates for further identification, when routine methods (MALDI-TOF-MS) cannot identify correctly to species level and isolates from hospital-acquired bacteraemia and central catheter line infections are included for outbreak detection. *Salmonella* isolates are included for serovar prediction, and Shiga toxin-producing *Escherichia coli* are included for serotype prediction and toxin gene typing.

Analysis results of sequences passing quality requirements are entered into the department’s LIS. Supplementary laboratory reports are sent out at the discretion of the clinical microbiology consultant following local instructions and a case-by-case evaluation. This will often consist of correcting a previously reported species identification obtained by routine methods or, e.g. reporting if the toxin subtypes identified in an STEC isolate are associated with haemolytic uremic syndrome. Information requiring specific knowledge and expertise to interpret, such as specific AMR genes, MLST or PlasmidFinder results, is not reported to the requesting clinicians.

Selected analysis results and metadata for all of the batch’s sequences, including QC pass/fail information, are imported into a custom MySQL database, so that sample numbers, file locations, etc. can be retrieved for outbreak investigations and other uses.

At our department, the pipeline is run on a regional compute cluster with 256 cores and 160 GiB of memory.

From October 2022 until late March 2023, the pipeline has been used to process sequencing data from 498 individual bacterial isolates in 30 analysis runs. Results from these analyses have been used in various clinical contexts.

In one case, the department of nephrology at OUH noticed an increase in *Staphylococcus aureus* central line-associated bloodstream infection (CLABSI) among patients receiving haemodialysis. DCM OUH was requested to investigate this as a potential outbreak. Nine isolates from samples requested from eight individual patients from the department of nephrology had been sequenced at that time. As CLABSI were included in the prospective routine sequencing and the RSYD-BASIC pipeline performed MLST typing by default, it was immediately possible to conclude that most samples requested from this department had different sequence types with a distance of multiple alleles to each other (as shown in Table 7).

**Table 7.** Count of ST types in *Staphylococcus aureus* samples from the suspected outbreak

ST type	Count
1	2
5	1
8	1
9	1
30	2
398	1

**Fig. 3.**
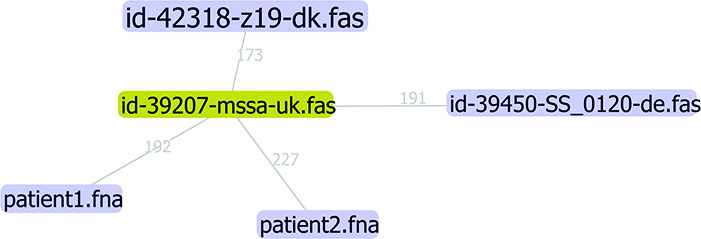
Minimum spanning tree of *Staphylococcus aureus* ST1 patient and background sequences based on cgMLST obtained with ChewBBACA.

This suggested that the majority of cases were not closely related. Two pairs of isolates showed the same sequence type, of which one pair originated from the same patient. Core genome MLST and SNP analysis could therefore be applied in a focused way to the remaining pair of isolates identified by RSYD-BASIC, and it was concluded that they were too distantly related to represent an outbreak – as shown in Fig. 3, both were closest to a ‘background’ strain in the minimum spanning tree based on cgMLST.

The applied approach to prospectively disprove the suspected outbreak permitted the department of nephrology to focus on improving patient-related infection control practices, rather than performing outbreak investigation and possible screening of personnel and environments [53]. This example highlights one aspect of the value of prospective WGS for surveillance purposes – the ability to disprove clonal outbreaks. Several reports have been published arguing the cost-effectiveness, clinical value and merit for infection control of implementing routine WGS of select bacterial isolates [54,57]. In a future version of RSYD-BASIC, automated phylogeny and screening reports for defined surveillance species may be added to support infection control efforts in real time.

## Conclusions

Routine sequencing can be a powerful tool in clinical microbiology, but the vast amounts of data it produces must be analysed to harness its power.

With RSYD-BASIC, we demonstrate a user-friendly open-source pipeline that, once set up by a bioinformatician, allows users without in-depth familiarity with the command line to obtain a broad range of clinically relevant results from bacterial isolate sequences.

When tested on publicly available data, RSYD-BASIC reached the same results as the original studies for most samples. However, in one case, a difference of a single nt led to a difference in MLST types; this serves to underline the importance of standardized workflows. Additionally, a comparison of CARD-RGI results for the larger dataset shows that full documentation of all settings is important to ensure full reproducibility. This can be difficult in manually run workflows. With a defined workflow controlled through a workflow management system, as is the case for RSYD-BASIC, commands and settings are documented in the workflow definition, making the workflow more reproducible.

Bioinformatic analysis is often one of the hurdles in implementing truly routine WGS of bacterial isolates. When such analyses can only be performed by bioinformatic experts, this is not only time-consuming but also carries the risk of one person’s absence or illness completely stopping the process. Training laboratory technicians to perform each individual analysis step manually alleviates the dependence on a single person but still risks human error at every step that requires manual entry, while occupying a laboratory technician for what may be a substantial time, especially as samples cannot easily be processed in parallel in this way. With a pipeline that can be routinely started by laboratory technicians, the laboratory workflow is faster and more robust. Additionally, bioinformaticians are able to spend more time on in-depth analyses that require their expertise, or on developing and extending bioinformatic tools, while laboratory technicians are available for routine diagnostic tasks. The range of information generated by RSYD-BASIC also provides us with a ‘head start’ in outbreak investigations, as more in-depth and computationally expensive analyses can be performed subsequently in a more targeted manner.

## supplementary material

10.1099/acmi.0.000646.v6 Supplementary Data Sheet 1.

10.1099/acmi.0.000646.v6 Supplementary Material 1.

10.1099/acmi.0.000646.v6 Supplementary Material 2.

## Abbreviations

AMR: antimicrobial resistance
CLABSI: central line-associated bloodstream infection
DCM OUH: Department of Clinical Microbiology, Odense University Hospital
LIS: laboratory information system
QC: quality control
SRA: Sequence Read Archive
ST: sequence type
WGS: whole-genome sequencing
