# Supplementary material for: RSYD-BASIC: a bioinformatic pipeline for routine sequence analysis and data processing of bacterial isolates for clinical microbiology
Source: Access Microbiol. 2025 Mar 21;7(3):000646.v6. doi: 10.1099/acmi.0.000646.v6 (PMC11927588; doi:10.1099/acmi.0.000646.v6)
Supplement: Supplementary Data Sheet 1. [file acmi-7-00646-s001.pdf]

Supplementary data: commands run

Manual outbreak investigation

cgMLST with chewBBACA:

Commandline:

```
chewBBACA.py PrepExternalSchema -i [path to original S. aureus schema fastas] \
 -o [path to chewBBACA S. aureus schema output directory] --cpu 6
```

```
chewBBACA.py AlleleCall -i [file of filenames] \
 -g [path to chewBBACA S. aureus schema output directory] \
 -o [allele call output directory] --cpu 6
```

```
chewBBACA.py ExtractCgMLST \
 -i [allele call output directory]/results_[date]/results_alleles.tsv \
 -o [clean cgMLST output directory] --t 0
```

After visualization with Phyloviz, SNP trees for potentially related samples and background samples were created with snippy:

```
snippy-multi [tab-separated file of sample IDs and filenames] \
 --ref [S. aureus SCAID OTT1-2021 sequence] --cpus 6 > s_aureus_snippy.sh
```

```
bash s_aureus_snippy.sh
```

Running CARD-RGI on RSYD-BASIC assemblies

CARD data were downloaded manually; subsequently, data were unpacked, the database loaded and sequences analyzed with rgi.

```
tar -xvf card-data.tar.bz2 ./card.json
```

```
rgi load --card_json card.json --local
```

```
for asm_file in [RSYD-BASIC output dir]/*/assembly/*.fna; \
    do rgi main -i $asm_file -o kma_seqs_rgi/$(basename $asm_file) \
    -n 6 --local; \
done
```
